# Traffic Marker?: Early Exposure to Air Pollution Associated with Childhood Asthma

**DOI:** 10.1289/ehp.118-a80b

**Published:** 2010-02

**Authors:** Laura Alderson

**Affiliations:** **Laura Alderson** has written for *EHP* since the 1990s. She is a freelance writer based in Raleigh, North Carolina, specializing in medicine, science, and high technology

Asthma is now the most common chronic disease for children and a major cause of emergency room visits, hospitalizations, and school absences, according to the World Health Organization. Now a large population-based study has shown an association between elevated exposure to air pollution *in utero* and during the first year of life and a higher risk of asthma in preschool-aged children **[*EHP*** **118:284–290; Clark et al.]**.

There are multiple known risk factors for developing asthma, including genetic factors, diet, and exposure to secondhand tobacco smoke and allergens. The speed with which the disease has risen in most developed and developing countries suggests environmental exposures probably play a prominent role. Although air pollution is known to worsen existing asthma, a succession of recent studies is building evidence for an additional association between exposure to traffic-related air pollution and initial onset of asthma in children.

Using a nested case–control study design, researchers looked at administrative and health care data for nearly 3,500 children born in southwest British Columbia, Canada, in 1999 and 2000 who were diagnosed with asthma by age 4 years. Each case was age- and sex-matched to 5 randomly chosen controls born in the same region and time period.

To estimate air pollutant exposures, the researchers mapped the residential history of each child against air pollution data obtained from regulatory monitoring, land use regression modeling, and proximity to stationary pollution sources and to roads. These metrics were used to calculate average exposures for the duration of the mother’s pregnancy and the child’s first year of life. Nine pollutant exposures were evaluated: carbon monoxide, nitric oxide, nitrogen dioxide, particulate matter (PM_10_ and PM_2.5_), ozone, sulfur dioxide, black carbon, and wood smoke.

The highest risk of asthma was associated with exposure to the traffic-related pollutants carbon monoxide, nitric oxide, nitrogen dioxide, and black carbon; lesser associations were seen with exposure to PM_10_ and sulfur dioxide, as well as with proximity to industrial point sources. Proximity to roads was not associated with increased risk, but only a small number of children resided near major roads in the study population. Associations between air pollution and asthma were generally greater in girls than in boys, although asthma was significantly more common in boys, consistent with other populations. The authors observe that other researchers also have reported stronger associations in girls, although the finding is not entirely consistent.

This is one of the few studies to examine the effect of *in utero* exposure on pediatric asthma risk. However, because of relatively high correlation between *in utero* and first-year exposures, the relative importance of these periods could not be discerned.

